# 586. Primary Care Utilization and Attachment Among Burn Survivors: A Population-based Matched Cohort Study

**DOI:** 10.1093/jbcr/irag033.359

**Published:** 2026-04-06

**Authors:** Darby Little, Elliott K Yee, Barbara Haas, Laura Rosella, Gemma Postill, Liisa Jaakkimainen, Stephanie A Mason

**Affiliations:** University of Toronto, Toronto, ON; University of Toronto, Toronto, ON; University of Toronto, Toronto, ON; University of Toronto, Toronto, ON; University of Toronto, Toronto, ON; University of Toronto, Toronto, ON; University of Toronto, Toronto, ON

## Abstract

**Introduction:**

Many burn survivors experience poor long-term outcomes, including reduced quality of life and greater mortality than the general population, yet post-discharge interventions to improve these outcomes remain underexplored. Given that access to primary care is associated with improved long-term health, we sought to characterize post-discharge primary care utilization among burn survivors to guide healthcare resource planning and support the long-term health of this population.

**Methods:**

This retrospective, population-based, matched cohort study identified adults who survived to discharge after major burn injury between 2010-2022. Burn survivors were matched with up to five uninjured individuals on age, sex, and a propensity score based on individuals’ medical and psychiatric comorbidity, rurality, immigration status, and socioeconomic characteristics. The primary outcome was rate of primary care provider (PCP) visits within two years post-discharge, and the secondary outcome was post-discharge PCP attachment. PCP visit rates were analyzed using a Generalized Estimating Equation (GEE) negative binomial regression, and PCP attachment was assessed using a GEE log-binomial model.

**Results:**

We identified 1925 burn survivors (median [IQR] age 47 [27] years, 73% male) and matched them to 9484 uninjured individuals. The mean PCP visit rate was higher in burn survivors (4.4 vs. 3.5 visits per person-year; RR = 1.29, 95% CI: 1.20–1.39). Burn survivors had more visits for mental health, while uninjured individuals had more visits for routine care (*p*<.001). PCP attachment was similar between burn survivors and controls (90% vs. 89%; RR = 1.01, 95% CI: 0.99–1.03).

**Conclusions:**

Burn survivors had greater primary care utilization than uninjured individuals in the two years after burn injury and had a greater proportion of visits related to mental health. Burn injury should be recognized as a chronic condition requiring sustained primary care engagement.

**Applicability of Research to Practice:**

Given both the complexity and rarity of burn injuries, post-discharge survivorship care models are needed to support PCPs with the management of long-term burn-related care.

**Funding for the study:**

Innovation Fund Grant.

